# Authors' reply

**Published:** 2010-04

**Authors:** Harminder Singh, N. Dulhani, Kumar Bithika Nel

**Affiliations:** Government Medical College, Jagdalpur, Bastar, Chhattisgarh, India

Sir,

This is with reference to the comments on our article.[1] The necessary clarifications are given as follows.

Hepatitis has been shown as an adverse drug reaction (ADR) to HAART, but some other studies do not report it commonly. We reported hepatotoxicity which includes hepatitis also.[2,3] Out of 79 patients, 68 had at least one ADR (reported) and 11 patients had no ADR at all. A total of 120 ADRs were reported since some patients had multiple ADRs. Out of 137 patients, 15 died due to terminal AIDS, 9 were lost to follow up due to change in address, etc, 18 were excluded from the study, and 16 were non-compliant (stopped therapy) and non-adherent to the follow-up protocol. Most severe ADRs were anemia and hepatotoxicity. Severity grading was classified according to the Hartwig and Siegel scale.[4] The correlation of ADRs with age, sex and CD4 count was done. Further analysis of difference in average age and CD4 count among males and females, though helpful, should not undermine the scope and information provided by our study.
